# Recent Advances in C–C Bond Formation via Visible Light-Mediated Desulfonylation and Its Application in the Modification of Biologically Active Compounds

**DOI:** 10.3390/molecules29235553

**Published:** 2024-11-25

**Authors:** Xiaohong Xu, Yufan Zhang, Xueyuan Zhang

**Affiliations:** 1 Chaozhou Institute for Drug Control, Chaozhou 521000, China; zhangyufan87@163.com (Y.Z.); snowdrop1122@163.com (X.Z.); 2 College of Pharmacy, Graduate School, Sun Yat-Sen University, Shenzhen 518107, China

**Keywords:** desulfonylation, photoredox, carbon–carbon bond formation, biologically active compound, radical

## Abstract

Developing efficient and novel methodologies to construct a C–C bond is highly important in both synthetic chemistry and pharmaceutical sciences. In recent years, the visible light-mediated desulfonylative transformation of sulfonyl compounds has emerged as a powerful tool for the synthesis of diverse C–C bond. To emphasize their practical utility, many methodologies have been successfully applied in the modification of a variety of biologically active compounds which possess unprotected amide or hydroxy groups. In this review, we would like to summarize recent advances in C–C bond formation via the visible light-mediated desulfonylation of sulfonyl chlorides, sulfinates, sulfonamides, sulfones, and sulfonylhydrazones. The reaction design, mechanism research, and the application of these protocols in the modification of biologically active compounds are presented. The challenges and future developments in this area are also discussed.

## 1. Introduction

The construction of the C–C bond is one of the most important and popular steps in synthetic chemistry [1]. In the last decades, significant achievements in the transition-metal-catalyzed functionalization of diverse C–X bonds, including C–H [2], C–O [3], and C–N [4], to forge C–C bonds have been obtained. The C–SO_2_ bond, which is usually not considered an applicable reaction partner because of its good stability, could also be functionalized in the presence of transition metals [5,6,7]. Therefore, sulfonyl compounds could serve as alternative good building blocks for the C–C bond construction via desulfonylation, which expands the chemist’s synthetic toolbox.

In recent years, visible light-mediated arylation and alkylation to synthesize the C–C bond draws extensive attention because these reactions usually could proceed under mild, environment-benign and transition-metal-free reaction conditions [8,9,10,11]. The desulfonylative transformations of sulfonyl compounds such as sulfonyl chloride, sulfinates, sulfonamides, and sulfones under visible light irradiation also have undergone significant improvements. Although several reviews have summarized the desulfonylative reactions from different aspects [12,13,14,15], in this review, we will focus on the visible light-mediated desulfonylation of diverse sulfonyl compounds to construct C–C bonds. Moreover, considering that biologically active compounds usually contain diverse unprotected groups, such as hydroxy, amine, and amide groups, using visible light can functionalize them without preprotection. Functionalization of these compounds suggests that these methodologies have excellent functional group tolerance and mild reaction conditions and possess a high potential for large-scale synthesis in industry and pharmaceutical synthesis. Therefore, the application of diverse desulfonylative reactions in the modification of biologically active compounds and their mechanisms are also presented in this review.

**Figure 1:**
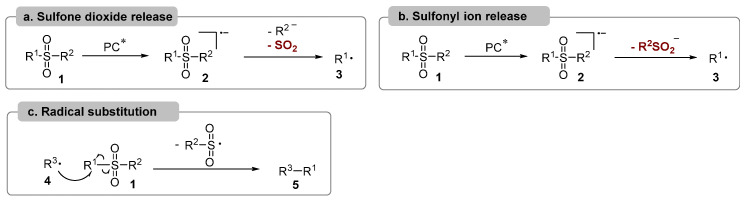
Mechanistic scenarios. PC* means excited photocatalyst.

There are three main mechanistic scenarios in the desulfonylative reactions [16,17,18] (Figure 1). The interaction between sulfonyl compound **1** and the visible-light-excited photocatalyst gives sulfonyl radical anion **2** via single electron transfer (SET). In this intermediate, if R^2^ is also a good leaving group, the release of anion species R^2^ and sulfone dioxide generate the R^1^ radical **3** which could produce further transformations to forge C–C bonds [18] (Figure 1a). And if R^2^ is not a leaving group, such as an alkyl group, the organic sulfonyl anion would be released to produce the R^1^ radical **3** [19] (Figure 1b). In another scenario, if the sulfonyl compound **1** could not be activated by excited photocatalyst directly, the radical species **4** generated from other process would attack the sulfonyl compound **1** to produce radical substitution to furnish product **5** by C–C bond formation and release the sulfonyl radical, which would be reduced to sulfonyl anion after interaction with another intermediate [20,21] (Figure 1c).

## 2. Sulfonyl Chlorides

**Figure 2:**
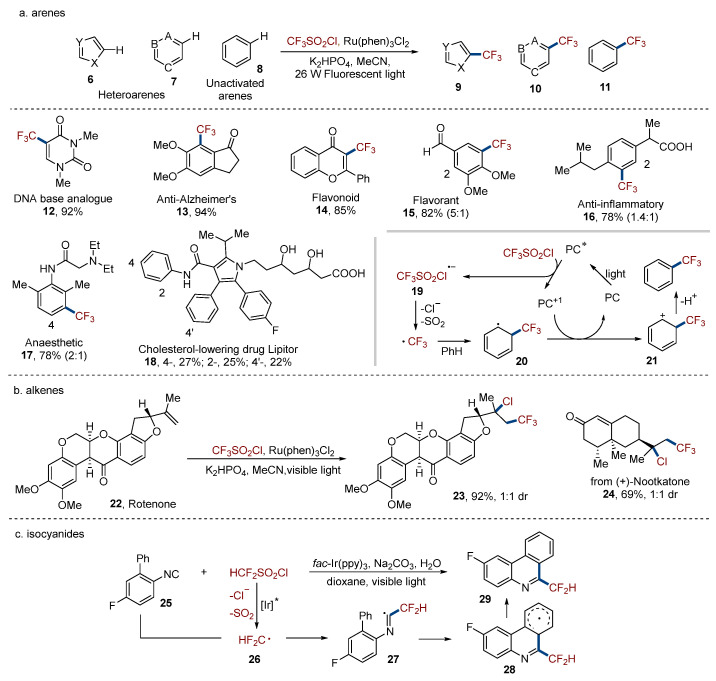
Sulfonyl chlorides. Ir* stands for excited iridium photocatalyst.

Sulfonyl chlorides are widely used as sulfonylation reagents via sulfonyl radicals generated by irradiation of visible light [22]. However, in the presence of fluoroalkylsulfonyl chlorides, desulfonylation usually happens to provide fluoroalkyl radicals, likely due to their good stability. In 2011, MacMillan [23] described the protocol of the desulfonylative trifluoromethylation of the C–H bond in diverse heteroarenes **6** and **7** and unactivated arenes **8** by photocatalysis (Figure 2a). With utilization of this method, diverse biologically active compounds could be functionalized in the position of high electron density and potential metabolic susceptibility. For example, DNA base analogue **12**, anti-Alzheimer’s drug **13**, and Flavonoid **14** could be trifluoromethylated regioselectively with a high efficiency (85–94% yield). For flavorant **15**, anesthetic **17**, and Lipitor **18**, a mixture of functionalization at different positions was obtained. The trifluoromethylation of the three positions at Lipitor **18** resulted in nearly 1:1:1 isomers. In this process, photocatalyst (PC) was excited by visible light and generated the excited photocatalyst (PC*). Then, the single-electron transfer (SET) of PC* and sulfonyl chloride provided the radical anion **19**. After the release of chloride anion and sulfone dioxide, a trifluoromethyl radical was produced and captured by arene to provide the intermediate **20**. Followed by oxidation and deprotonation, the trifluoromethylated product was obtained.

In this catalytic cycle, the trifluoromethyl radical can also react with alkenes and result in a cationic intermediate which is analogous to **21** after oxidation. If this cationic intermediate is captured by another anion, such as halide, other than deprotonation, a di-functionalization method is presented. Han [24] reported the chlorotrifluoromethylation of alkenes with CF_3_SO_2_Cl in the presence of photocatalyst under very simple reaction conditions (Figure 2b). Rotenone **22** and Nootkatone were difunctionalized to give chlorotrifluoromethylated products **23** and **24** in good- to high-efficiency with 1:1 diastereoselectivity by using this method. Besides this success, Dolbier and Reiser [25,26,27,28] also developed several methodologies for the difunctionalization of alkenes via visible light-mediated desulfonylation of sulfonyl chlorides.

Difluoroalkyl sulfonyl chlorides could also produce desulfonylation under visible light irradiation to generate an alkyl radical. Dolbier [29] proved that isocyanide **25** was a good reaction partner to react with the difluoroalkyl radical **26** derived from desulfonylation of sulfonyl chlorides and go through tandem addition and cyclization/oxidation to result in substituted phenanthridine **29** (Figure 2c).

## 3. Sulfinates

**Figure 3:**
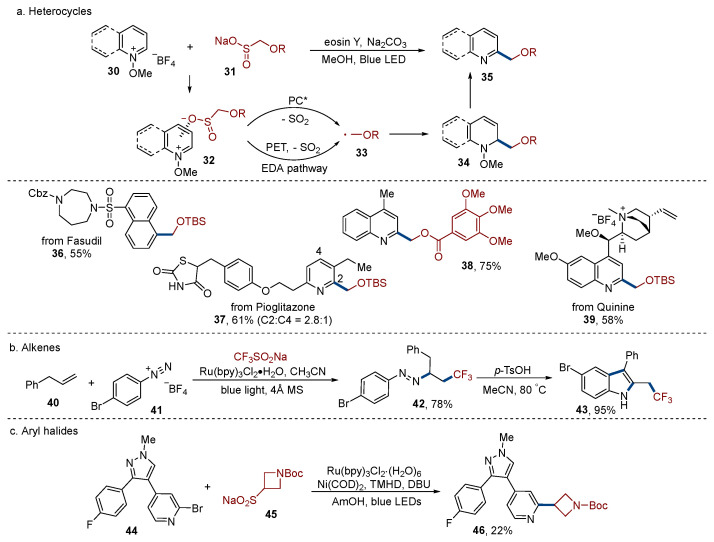
Sulfinates.

Sulfinates, which are also usually considered sulfonylation reagents [22], can go through desulfonylation to act as an alkyl radical precursor. Moschitto [30] found that alkyl sulfintes could produce the desulfonylative alkylation of heteroarenes under visible light irradiation (Figure 3a). Fasudil, pioglitazone, and quinine could all be alkylated to afford a modified product **36**–**39** in moderate efficiency. The unprotected amide (**37**), alkene (**39**), and ammonium (**39**) groups remained intact under these reaction conditions, revealing the good functional group tolerance of this methodology. Interestingly, the authors found that this reaction could also happen in the absence of light. Therefore, the authors believed that two mechanisms might be involved in this reaction. Firstly, an EDA (electron donor–acceptor) complex **32** was formed via ion exchange. Then, the desulfonylation reaction occurred to generate an alkyl radical **33** through either the interaction of this EDA complex with photoexcited catalyst or direct PET in EDA complex. The rapid addition of an alkyl radical into the heteroarene and subsequent oxidation process provided the final product **35**.

Sodium triflates are widely used as trifluoromethyl radical precursors to react with diverse radical acceptors [31,32]. Xiao [33] reported that sodium triflate reacted with alkene **40** and aryl diazonium **41** under visible light irradiation, generating trifluoromethylated azo product **42** in 78% yield (Figure 3b). This azo compound could be conveniently converted to trifluoromethylated indole **43** in 95% yield through a sequential isomerization and cyclization cascade in the presence of acid.

The cross-coupling of aryl halides and alkyl radicals is challenging, since usually photocatalyst/transition metal dual catalysis is required in the catalytic cycle. Knauber et al. [34] reported that the cross-coupling of alkyl sulfinate salts and aryl halides constructs the C_sp_^2^–C_sp_^3^ bond via Ru/Ni dual catalytic desulfonylation (Figure 3c). Diverse aryl and heteroaryl halides could react with a variety of alkyl sulfinate salts in moderate to good yields. The casein kinase 1*δ* inhibitor analogue **44** was alkylated to give **46** in 22% yield under the standard reaction conditions. Using this protocol, a small compound library could be built based on the pharmacophore, which highlighted the potential utility of this methodology in the drug discovery.

## 4. Sulfonamides

**Figure 4:**
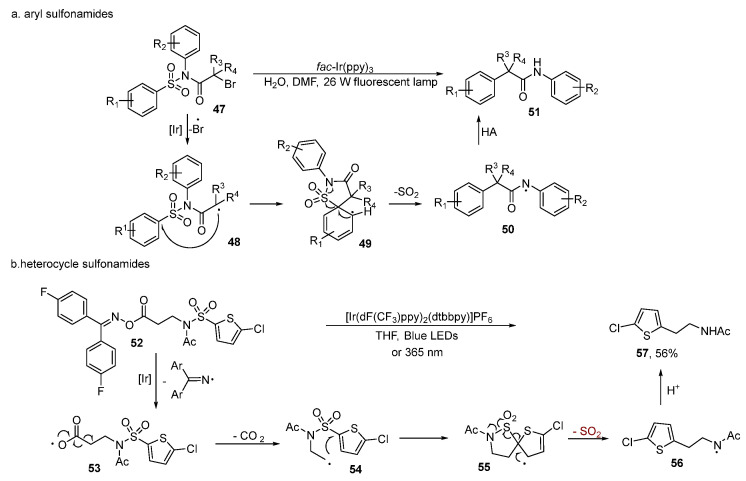
Sulfonamides.

The N–S(O_2_) bond in sulfonamides is quite stable. Therefore, the desulfonylative functionalization of sulfonamides usually occurs via the Smiles rearrangement initiated by various radicals [35,36,37,38]. In 2016, Zhang [39] reported a highly efficient intramolecular selective aryl migration and desulfonylation of sulfonamides via visible light-induced photoredox catalysis. On the basis of the experimental results, they proposed a plausible mechanism (Figure 4a). The iridium catalyst was first excited under visible light irradiation. Then a single electron transfer process was undergone with sulfonamides **47** to provide a radical. After the 5-ipso cyclization and rapid desulfonylation took place to generate a radical, the target product was obtained from hydrogen abstraction (HA).

In 2020, Duong and Greaney [40] described a method to construct the C_sp_^2^–C_sp_^3^ bond through visible light-mediated N–O bond cleavage, decarboxylation, and desulfonylative Smiles rearrangement (Figure 4b). Based on the previous references [41,42,43,44,45], the authors believed that an energy transfer (EnT) event, other than single electron transfer (SET), between the excited iridium catalyst and the imine happened to activate the N–O bond, generating the carboxyl radical **53** by homolytic N–O bond cleavage. After decarboxylation and Smiles rearrangement, a spirocyclic intermediate **55** was produced. Finally, the release of sulfone dioxide resulted in the product **57**. Chiral amines could be employed in this reaction and maintained optical purity, indicating the potential utility of this method in the synthesis of pharmaceuticals. Furthermore, when this reaction was irradiated under 365 nm UV light, the formation of the product could also occur without any catalyst.

## 5. Sulfones

**Figure 5:**
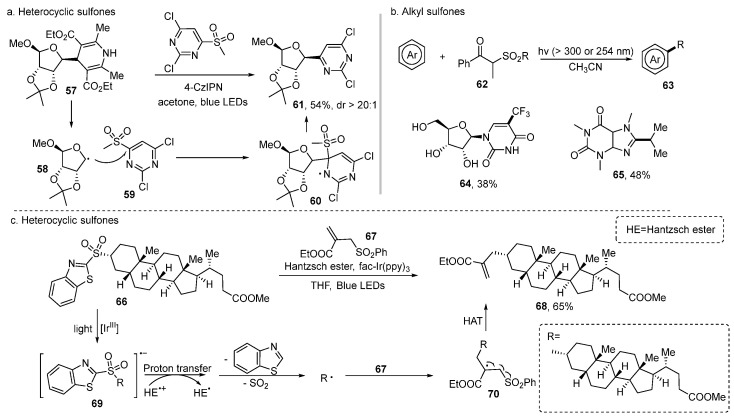
Sulfones.

Sulfone is another kind of stable sulfonyl compound. The C–SO_2_ bond in heterocyclic sulfones could be activated to produce desulfonylation to construct the C–C bond. In 2019, Molander [20] found that alkyl radicals resulted from alkyl bis (catecholato) silicates or 1,4-dihydropyridines (DHPs) could attack *N*-heteroaryl sulfones to furnish diverse alkylated heterocycles (Figure 5a). This protocol could be applied to saccharide scaffolds **57** which are valuable in drug discovery to produce heterocycle-attached saccharide motifs **61** in 54% yield while maintaining the diastereoselectivity excellently. The key step is that the alkyl radical derived from a single electron transfer (SET) from **57** to catalyst engages sulfone **59** to provide the intermediate **60**. Then, a sulfonate extrusion generates the final product **61**.

Li [46] found that alkyl sulfone **62** could act as a good alkyl radical precursor under UV light irradiation (Figure 5b). Nucleoside could be trifluoromethylated to furnish products **64** in 38% yield under this redox-neutral reaction conditions without any catalyst. While using 254 nm irradiation, general nucleophilic alkyl radical could also be generated from the precursor **62** and could be installed on the electron-deficient heteroaromatics. For example, the isopropyl group attached caffeine **65** could be obtained in 48% yield with using this methodology.

Benzothiazole alkyl sulfones also have been proven to be good alkyl radical precursors. Chatterjee [47], Yoda [48], and Hu [49] found that visible light also can activate these sulfones to generate alkyl radicals and proceed a variety of subsequent transformations to construct C–C bonds. In 2022, Chatterjee [47] developed a protocol to forge the C_sp_^3^–C_sp_^3^ bond via double desulfonylative steps and successfully applied to functionalize lithocholic acid ester compound **66** (Figure 5c). In this reaction, SET event between **66** and photocatalyst occurred to furnish the radical anion intermediate **69**. Then, fragmentation of this species produced an alkyl radical along with the release of sulfone dioxide and benzothiazole. The subsequent capture of this radical by alkene **67** resulted in intermediate **70**. Radical elimination provided the final product **68**.

## 6. Sulfonylhydrazones

**Figure 6:**
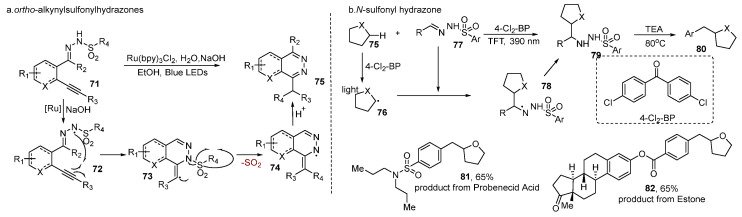
Sulfonylhydrazones.

Sulfonylhydrazone is another kind of sulfonylation that could easily go through desulfonylation via Smiles rearrangement under visible light. In 2016, Belmont [50] found that *ortho*-alkynylsulfonohydrazone precursors could be active under visible light to synthesize various phthalazines with high yield (Figure 6a), which involved a radical hydroamination reaction followed by a radical Smiles rearrangement. Phthalazine structures are found in many important biological relevant compounds’ possessing properties, such as antibacterial [51] or antitumo [52] agents. Based on the observations of several control experiments and measurements, a plausible mechanism was proposed. Sulfonohydrazone is first deprotonated in MeOH, which is then photo-oxidized by the excited ruthenium-based photocatalyst to generate radical **71**. The triple bond was attacked by the radical. After Smiles rearrangemet and decarboxylation, phthalazine was produced.

In 2023, Pulcinella and his group [53] reported the alkyl–alkyl cross-coupling reaction, which combines N-sulfonyl hydrazones and C(sp3)–H donors through a diarylketone-enabled photocatalytic hydrogen atom transfer (Figure 6b). This metal-free protocol provides a useful way to create various medicinally relevant compounds, such as homobenzylic ethers, aryl ethyl amines, and *β*-amino acids. In this reaction, trifluoromethyl-phenylsulfonyl hydrazones derived from drugs and natural products, such as probenecid acid **81**, estrone **82**, were successfully engaged as coupling partners with good yield. Upon the absorption of a 390 nm photon, excited 4-Cl_2_-BP cleaves the C-H bond in the cycloalkyl group to generate nucleophilic alkyl radical **76**. This transient species can undergo a polarity-matched addition to the electrophilic site of **77**, delivering a putative hydrazinyl radical **78**. Then **78** was reduced by the reduced form of 4-Cl_2_-BP and generated the Csp3–H alkylated intermediate product **79**. After the complete photocatalytic cycle, triethylamine (TEA) was added directly to the reaction system to break **79** down and ultimately yielded the targeted cross-coupled product **80**.

## 7. Conclusions and Outlook

In this review, the construction of C–C bonds by visible light-mediated desulfonylation of sulfonyl chlorides, sulfinates, sulfonamides, and sulfones have been summarized. These synthetic systems usually possess the advantages of mild reaction conditions and good efficiency. The application of these methodologies on the modification of biologically active compounds suggested the wide substrate scopes and potential utilization in the industry. However, many challenges also remain. Firstly, the substrates which are able to produce desulfonylation are limited to several types of active compounds, such as fluorinated or heterocyclic sulfonyl compounds, and this transformation of simple alkyl and aryl sulfonyl substrates still needs to be explored. Secondly, sulfonyl compounds such as sulfonyl chlorides and sulfinates are usually used as sulfonylation reagents. The developments of catalytic systems to selectively control the sulfonylation and desulfonylation pathways is of great significance; however, it is rare to the best of our knowledge. It is reported that scientists have used photoenzymes to catalyze asymmetric radical C–C couplings [54]. As nature’s privileged catalysts, enzymes have unparalleled advantages in asymmetric synthesis. With the improvements of synthetic chemistry and the utilization of photoenzymes in this field in the future, selective control of sulfonylation and desulfurization could be achieved soon. We believe that more desulfonylative transformations will be developed to prosper the research in this field, which proves desulfonylation to be one of the most important synthetic tools in synthetic chemistry and pharmaceutical sciences.
